# The Combined Influence of Air Pollution and Home Learning Environment on Early Cognitive Skills in Children

**DOI:** 10.3390/ijerph14111295

**Published:** 2017-10-26

**Authors:** Elle Lett, Jeanette A. Stingone, Luz Claudio

**Affiliations:** 1 Perelman School of Medicine, University of Pennsylvania, Philadelphia, PA 19104, USA; lanair.lett@uphs.upenn.edu; 2 Department of Environmental Medicine and Public Health, Icahn School of Medicine at Mount Sinai, New York, NY 10029, USA; luz.claudio@mssm.edu

**Keywords:** air pollution, school readiness, isophorone, ECLS-B, child development, neurodevelopment

## Abstract

Cognitive skills are one component of school readiness that reflect a child’s neurodevelopment and are influenced by environmental and social factors. Most studies assess the impact of these factors individually, without taking into consideration the complex interactions of multiple factors. The objective of this study was to examine the joint association of markers of environmental pollution and of social factors on early cognitive skills in an urban cohort of children. For this, we chose isophorone in ambient air as a marker of industrial air pollution. Low quality home learning environments was chosen as a marker of the social factors contributing to cognitive development. Using a subpopulation from the Early Childhood Longitudinal Study, Birth Cohort (N = 4050), isophorone exposure was assigned using the 2002 National Air Toxics Assessment. Home learning environment was assessed with a modified version of the Home Observation for Measurement of the Environment (HOME) Inventory, and standardized math assessment scores were used as a measure of early cognitive skills. Multiple linear regression was used to estimate the effect of both exposures on math scores. After adjustment for confounders, children living in areas with ambient isophorone in the upper quintile of exposure (>0.49 ng/m^3^) had math scores that were 1.63 points lower than their less exposed peers [95% CI: −2.91, −0.34], and children with lower HOME scores (at or below 9 out of 12) had math scores that were 1.20 points lower than children with better HOME scores [95% CI: −2.30, −0.10]. In adjusted models accounting for identified confounders and both exposures of interest, both high isophorone exposure and low HOME score remained independently associated with math scores [−1.48, 95% CI: −2.79, −0.18; −1.05, 95% CI: −2.15, 0.05, respectively]. There was no statistical evidence of interaction between the two exposures, although children with both higher isophorone exposure and a low HOME score had a decrement in math scale score beyond the additive effect of each exposure. This was primarily observed among male children. These findings suggest that aspects of both the physical and social environments are independently associated with children’s early cognitive skills. Future research aiming to improve children’s early cognitive skills and subsequent school readiness should address both domains.

## 1. Introduction

School readiness consists of the behavioral, social, and cognitive skills that determine a child’s ability to successfully learn when entering the school system [1]. Several studies have demonstrated that various sociodemographic factors influence school readiness including household income, race, and family structure [2]. Because school readiness is an early predictor of children’s academic achievement, there is great interest in identifying modifiable factors that are associated with children’s early cognitive skills that can then serve as a target for interventions [3,4]. A component of school readiness [1], early cognitive skills, can reflect a child’s neurodevelopment and are affected by both social factors and aspects of the physical environment including environmental pollution.

Specifically, parenting behaviors that can promote or detract from early learning and development have been found to be important in influencing early cognitive skills. Nurturing parent-child interactions characterized by affection [5], limited criticism [6], and child freedom [7] are associated with school readiness and subsequent academic achievement later in life. Also, specific parenting practices, including reading to children and using complex language, have been shown to promote development of specific early cognitive skills like reading and numeracy [8]. There is also a growing literature on the effect of environmental factors, such as exposure to air pollutants, on neurodevelopment in children [9]. Studies have found associations between ambient levels of air toxics and lower grade point averages in elementary school [10]. There are also studies which observe associations between exposure to air pollutants prenatally and behaviors or symptoms later in childhood that are suggestive of problems with academic success. For example, Fuertes et al. found an association between exposure to particulate matter prenatally and hyperactivity/inattention at age 15 [11].

Despite the evidence that both social and environmental risk factors influence child development, few studies have examined the combined effects of these classes of exposures. In their review of risk factors contributing to poor developmental outcomes in children, Walker et al. posited that psychosocial and biological factors related to poverty drive outcome disparities in cognitive, sensory-motor, and social-emotional development for disadvantaged populations. They identified caregiver-child interactions that influence learning and environmental toxicants among many other factors that jointly contribute to health inequities. This highlights the need to view risk factors holistically, examining how combinations of exposures that cluster in specific subpopulations may have additive or synergistic effects [12,13].

The present study aims to examine the joint association of a marker of environmental pollution and a marker of the social environment on early cognitive skills in an urban subset of the Early Childhood Longitudinal-Study Birth cohort (ECLS-B). For this, we chose isophorone in ambient air as a marker of industrial air pollution. Isophorone is a volatile organic compound that is commonly used as a solvent in inks, paints, coatings, and adhesives and is released into ambient air surrounding industrial facilities that either produce or use these solvents [14]. The printing industry is one example of an industry with historical exposures to isophorone. There have not been previous studies to understand the effect of perinatal exposure to isophorone on early cognitive skills or school readiness, though human occupational hazard studies have shown that isophorone exposure can have neurological effects such as dizziness and fatigue in adults exposed in the workplace. Additionally, measures of total organic solvent use have been associated with neurobehavioral outcomes in exposed workers within the printing industry [15]. A data-driven study of over 100 air toxics identified isophorone as a marker of a pollutant profile associated with children’s school readiness in previous research within this study population [16]; isophorone was thus selected to serve as a marker of industrial pollution within this study. Also, we used low quality home learning environment as a marker of the social factors that may be involved in neurodevelopment. As a proxy for cognitive skills, we used scores on a standardized math assessment designed to measure readiness to enter kindergarten in children [17]. We focused on math test scores because previous research has found that mathematics skills at school-entry are highly predictive of later academic achievement [18].

## 2. Materials and Methods

### 2.1. Study Population

The Early Childhood Longitudinal Study-Birth (ECLS-B) cohort is a nationally representative sample of approximately 10,700 children born in 2001 and followed through kindergarten as part of the Early Childhood Longitudinal Study sponsored by the National Center of Education Statistics (NCES). The purpose of the birth cohort was to study how characteristics of early home environments relate to development and kindergarten readiness. Data on family demographics, early child care, preschool, and school environments were collected across study waves at 9 months after birth, 2 years after birth, and preschool and kindergarten ages (4–6 years of age), through a series of parent, care provider, and teacher surveys and interviews. Additionally, in-home observations of semi-structured tasks were conducted at the preschool wave to assess the nature of the parent-child relationship [17].

For the purpose of this study, the analyses were restricted to those children who were followed from enrollment in the ECLS-B through kindergarten and, therefore, had measurements for the outcome of interest, math scores (N = 8900). The analytic cohort was subsequently restricted to those children who had a residential address provided during the baseline visit and had complete data for all confounders (N = 5550). Lastly, we further restricted the analysis to include only those children residing in urban areas at the time they were enrolled in the ECLS-B (N = 4050). Urban areas were defined in the ECLS-B based on minimum population density requirements and overall populations greater than 50,000 people as described by the 2000 Census criteria [19,20]. We chose to focus our analyses on children in urban environments because these settings represent the greatest risk for high air pollutant exposure [21]. Throughout this manuscript, all population counts are rounded to the nearest 50 in accordance with NCES restrictions on ECLS-B data presentation. This study was reviewed and approved by the NCES and the Institutional Review Board of the Icahn School of Medicine at Mount Sinai (HS#14-00230).

### 2.2. Outcome Assessment

Early mathematics skills were evaluated using a 58-item assessment derived from a pool of 71 questions and administered to children as part of the kindergarten wave of the ECLS-B. This assessment included questions that measured cognitive skills related to school readiness in mathematics across several categories including, number sense, spatial sense, data analysis, measurement, and patterns [17]. Of note, two sets of measurements on the kindergarten wave of the ECLS-B were obtained, one for children entering kindergarten in the 2006–2007 academic year, and one for children entering or repeating kindergarten in the 2007–2008 academic year. For the purpose of this study, only the first math scale score was used for children who repeated kindergarten.

### 2.3. Exposure Assessment

Isophorone exposure levels were determined by linking the ECLS-B database with the 2002 National Air Toxics Assessment (NATA). NATA is a periodic assessment conducted by the Environmental Protection Agency (EPA) to estimate the ambient air concentration of non-criteria air pollutants across the US. The estimation process combines data from known emission sources with advanced computational models to produce estimates of air pollutant concentrations at the census-tract level [22]. Isophorone exposure for each child was assigned based on ambient concentrations estimated for the ZIP Code of the primary residence at study enrollment (i.e., age 9 months). Because the NATA assessments provide census-tract specific estimates of isophorone concentrations, weighted average exposures for each ZIP Code were constructed using the percent of residential housing within each ZIP Code that lies within each census-tract. These data were obtained from the Housing and Urban Development and United States Postal Service ZIP Crosswalk files. This method is similar to approaches used in previous studies linking NATA to ECLS-B data [23]. The distribution of isophorone was right-skewed, with the upper quintile representing the majority of the range of exposure levels. Therefore, isophorone was dichotomized for exposure levels greater than or equal to the 80th percentile (0.49 ng/m^3^).

The second exposure, home environment, was measured in the ECLS-B study using an adapted version of the Home Observation for Measurement of the Environment Inventory Short Form (HOME-SF) [24]. The HOME Inventory is a validated instrument, designed to measure the quality of the home learning environment based on a combination of observations and parent interviews [25]. Measures from the HOME Inventory have been associated with neurodevelopment, physical health, and language competence, and have been used reliably across economically and ethnically diverse populations [26].

Consistent with previous research, HOME scores were calculated based on characteristics of the parent-child interaction identified during the observation or ascertained from parent interviews conducted during the second wave of the ECLS-B. Positive behaviors that were identified during observation included: (1) Spontaneously speaking to the child (excluding scolding); (2) verbally responding to the child’s speech; (3) physical displays of affection; (4) providing toys or activities for the child; (5) keeping the child in view; (6) and providing a safe play environment for the child. Negative parent behaviors included: (7) Spanking or slapping the child and (8) interfering or restricting the child’s environment. Additional behaviors that were identified from the parent interviews included: (9) reading books to the child; (10) allowing the child to accompany when running errands; (11) singing songs with the child; and (12) telling stories to the child. All measures were dichotomized and after reverse-coding the negative behaviors, the sum across all measures was used as the final HOME score, for a scale that ranged from 0 to 12. The distribution of HOME scores was left-skewed, so the exposure was dichotomized at a score of 9. This marked the lower 30% of the HOME score distribution, and individuals with a score of 9 or lower were treated as exposed to lower quality home learning environments than children with HOME scores greater than 9.

### 2.4. Confounders

Confounders were selected a priori based on known associations with academic performance outcomes and exposure to air pollutants established in the literature [27,28]. These confounders included child race, maternal age at childbirth, maternal marital status at childbirth, primary language spoken in home, and socioeconomic status (SES). The child demographic information was obtained at enrollment in the ECLS-B cohort and the maternal age and marital status at childbirth were derived from birth certificates and reported in the ECLS-B database. Primary language was based on the language spoken at home at the 2-year wave of the study. A marker for recent immigration, individuals whose primary language was not English may have different environmental exposures due to the residential location of immigrant neighborhoods [29]. This would need to be adjusted separately from measures of SES. We used a binary indicator that collapsed all non-English languages into a single category for this confounder. Child race was categorized as Black (Non-Hispanic), White (Non-Hispanic), Asian, and Hispanic, with all other races collapsed into an “Other” category because of their comparatively low frequencies in the study population.

The effect of SES was controlled by using quintiles of a continuous index developed by the ECLS-B that incorporated parent education, occupation, and household income [19]. Finally, a neighborhood deprivation index (NDI) was calculated and linked to each child by ZIP Code of primary residence at study enrollment. The NDI is a measure that summarizes neighborhood level disadvantage derived from principal component analysis across several sociodemographic indicators that have been linked to various health outcomes [30]. The following U.S. Census based-factors were used to construct the NDI: percent of males in professional occupations, percent of males in management occupations, percent of households with a female head of household, percent of households with residential crowding, percent living in poverty, percent receiving public assistance, percent unemployed, percent of adults with less than a high school education, percent of residents under the age of 35.

### 2.5. Statistical Analyses

Summary statistics for the overall cohort and subpopulations based on exposure status were calculated in R. Multiple linear regression was used to model the relationship between math scores and both exposure measures (i.e., estimated exposure to isophorone in ambient air and markers of low quality home learning environments). Models were constructed to examine individual main effects, additive effects (i.e., including both exposures in a single model), and modification between the two exposures. Modification was assessed by including an interaction term between the two exposures in the model and then assessing its statistical significance with a Wald test, using an alpha level of 0.1. All regression analyses accounted for correlation and unequal weighting due to clustering and oversampling in the ECLS-B survey design. Models were built with PROC SURVEYREG in SAS^®^ 9.3 (SAS Institute, Cary, NC, USA), using Taylor series linearization to estimate the standard errors for parameter estimates [31]. Results are reported as crude and adjusted parameter estimates with corresponding 95% confidence intervals (CIs). Model estimates for confounding variables are not presented, as our models were built to estimate the adjusted effect of isophorone and home environment on school readiness.

## 3. Results

Among the 4050 children in the study cohort, math scores ranged from 11.13 to 69.69 with a mean and standard deviation of 41.89 and 10.91, respectively. Eight hundred of the children were in the upper quintile of isophorone exposure, with exposures greater than 0.491 ng/m^3^ (Table 1), and comprised the high exposure group. Children with high isophorone exposure were more likely to be Black or Hispanic than those with low exposure, while children with low exposure to isophorone were more likely to be White or Asian. Children with high isophorone exposure were also more likely to have a language other than English as the primary language spoken at home, have a lower socioeconomic status, be born to younger mothers, and be born to unmarried mothers. In addition, children with high isophorone exposure were more likely to live in ZIP Codes linked to higher NDIs, indicating residence in disadvantaged neighborhoods.

For the home learning environment exposure, children with HOME scores less than or equal to 9, were considered “exposed” to a low-quality home learning environment (N = 1250, 30.9%). Low HOME score exposure showed similar associations with all confounders as high isophorone exposure (Table 1), with children in that group more likely to be Black or Hispanic, have a lower socioeconomic status, have younger mothers, unmarried mothers, and come from more disadvantaged neighborhoods.

In the unadjusted analysis, children with high isophorone exposure had, on average, math scores that were 3.81 points lower than those of children with low isophorone exposure (95% CI: −5.12, −2.51, Table 2). This effect estimate remained after adjustment for confounders, with children with high isophorone exposure scoring an average of 1.63 points lower (95% CI: −2.91, −0.34, Table 2). We also observed a relationship between HOME score and math scores in unadjusted analyses with children in the low HOME score exposure group having an average of 3.06 points lower than those with higher HOME scores (95% CI: −4.24, −1.89, Table 3). This estimate was also present after adjustment (−1.20, 95% CI: −2.30, −0.10, Table 3).

Unadjusted models that included isophorone and home learning environment showed significant associations between both exposures and math scores (Table 4). After adjusting for confounders (Table 4), both high isophorone exposure [−1.48, 95% CI: −2.79, −0.1] and lower HOME score [−1.05, 95% CI: −2.15, 0.05] were associated with decreased math scores and the magnitude of their respective associations was only slightly attenuated when compared to the results of their individual models.

The potential for effect measure modification between the two exposures was investigated by adding an interaction term between isophorone exposure and HOME scores to the unadjusted and adjusted models (Table 5). There were approximately 350 children (8.3%) who were exposed to both high isophorone concentrations and low quality home learning environments. Our results suggest they experienced a decrement in math scale score beyond the additive effect of both exposures, though this effect was not statistically significant in our analysis (−0.92, *p*-value = 0.41). Interestingly, this modification appears to differ by gender, with only male children showing the additional decrease [−2.37, 95% CI: −5.87, 1.12] in math scale scores in relationship to dual exposure (Table 5). However, the interaction with gender was not statistically significant and should be cautiously interpreted.

## 4. Discussion

In the present study, we observed that there were associations between decreased performance on math assessments and both early-life isophorone exposure and low HOME score. Inclusion of an interaction term in the overall adjusted model was not statistically significant. Interestingly, children with both higher isophorone exposure and a low HOME score had a decrement in math scale score beyond the additive effect of each exposure. This was especially pronounced among male children, where the estimated effect of the interaction between isophorone exposure and low HOME score was a two-point decrement in test score, though this result was not statistically significant. These findings suggest that future studies aimed at understanding children’s early cognitive skills should address the adverse effects of both the physical and social aspects of a child’s environment.

Our results are consistent with a previous study that examined another aspect of the early childhood environment, economic hardship, in relation to air pollutant exposure. In that study, Vishnevetsky et al. showed that prenatal exposure to polycyclic aromatic hydrocarbons (PAHs) had a negative effect on child IQ, but only among children born to mothers who experienced severe material hardship during pregnancy or immediately thereafter [32]. Our work investigates a similar hypothesis, that exposure to an air pollutant and the effects of sociodemographic factors can both affect neurodevelopment and influence children’s school readiness, as measured by their early cognitive skills.

The interaction between isophorone exposure and home environment appeared to differ by gender based on the stratified analysis, suggesting that a lower quality home environment is more detrimental to academic readiness in young males than young females similarly exposed to high ambient concentrations of isophorone. This is consistent with a previous study that showed that parental nurturance, an aspect of the home environment, had a greater positive effect on child working memory in males than in females [33]. That same study also found an interaction between chlorpyrifos exposure, a neurotoxic pesticide, and gender on working memory, with chlorpyrifos exposure in males being associated with greater deficits in working memory than in females [33]. Although our findings did not reach statistical significance, the magnitude of the difference in effect estimates coupled with findings from previous research suggest that there may be a complex relationship between social environment, pollutant exposure, and gender which requires further study.

There are several limitations to this study. Firstly, the spatial and temporal constraints of the NATA estimation process may lead to misclassification with respect to isophorone exposure, as has been described in previous studies using this data source [28]. Specifically, because NATA is a periodic assessment, the closest estimates for perinatal exposure for the 2001 birth cohort was the 2002 NATA, which may not be indicative of isophorone exposure during critical windows of susceptibility to pollutant toxicity. Further, the home address provided in the study was the location of the child at 9 months of age. There was no information on residential mobility during the perinatal and early-life period that could also affect exposure misclassification. Because the isophorone metric used in this study is the result of a multi-stage modeling process, there are multiple uncertainties and potential opportunities for error within the estimation process. These limitations in exposure assessment contribute to non-differential misclassification, likely causing an underestimation of the relationships studied. The limited monitoring data available for air toxics, such as isophorone, necessitates the use of these modeled exposures when examining nationally representative samples.

Currently, little is known about the potential biological effects of isophorone exposure. It is unclear based on our present analyses whether isophorone is causal in the observed deficits in neurocognitive development, or if it is only associated with other neurotoxicants that drive the effect. For example, lead is a well-established neurotoxicant that often varies spatially. If lead covaries with isophorone, then the inability to adjust for lead within this study could lead to residual confounding. As written in the introduction, it is likely that isophorone is a marker for industrial activity that emits solvents and other environmental pollutants into the ambient air. Further studies that incorporate mediation analyses and parse the complex relationship of isophorone with other pollutants and their effects on neurodevelopment are necessary to fully characterize the relationship observed here.

Another limitation may be that the modified version of the HOME Inventory used in this study represents a subset of the original HOME Inventory [19]. As with previous studies using this version of the HOME Inventory included in the ECLS-B, we accounted for the reduced resolution of the HOME Score, as well as the skewness of the distribution of scores, by dichotomizing the variable to categorize home learning environments [34]. This approach may have decreased the precision of our estimates, contributing to the lack of statistical significance of the additive effects and the interaction between air pollution exposure and home environment. Although it represented the lower 30% of the distribution, the group of children with “low” HOME scores consisted of children with scores as high as 9 out of 12. The small number of individuals with low HOME scores reduced the contrast between the two groups, and potentially contributed to misclassification. Similar to the misclassification of air pollution exposure, this potential misclassification of home environment is likely non-differential and would result in underestimating the association between low HOME scores and children’s math scores. Despite these limitations, it was still possible to observe associations between air pollution, home environment, and math scores. However, examining this research question in larger cohorts with more resolved measures of environmental exposures and home learning environment as well as populations with greater exposure contrasts could assess this relationship more conclusively.

## 5. Conclusions

This study demonstrates that both early childhood exposure to ambient isophorone and low quality home learning environments are independently associated with decreased performance on assessments measuring cognitive skills necessary for school readiness in mathematics. Further, this work is part of a growing body of literature targeted at understanding how environmental pollutants and social determinants of health collectively influence neurobehavioral outcomes in children [35]. Future research aiming to improve children’s early cognitive skills and advance their school readiness should consider the impacts of factors in both environmental and social domains, particularly when investigating outcomes in urban populations.

## Figures and Tables

**Table 1:** Sociodemographic characteristics by level of exposure to isophorone in ambient air and Home Observation for Measurement of the Environment (HOME) Score among the subset of the Early Childhood Longitudinal-Study Birth cohort (ECLS-B) 2001 living in urban communities. SES = socioeconomic status.

Characteristic	All (N = 4050), %	Isophorone > 0.49 ng/m^3^ (N = 800), %	Isophorone ≤ 0.49 ng/m^3^ (N = 3200), %	HOME Score ≤ 9 (N = 1250), %	HOME Score > 9 (N = 2800), %
Gender					
Female	49.83	50.87	49.57	49.16	50.13
Male	50.17	49.13	50.43	50.84	49.87
Race					
White	37.92	26.55	40.76	27.43	42.62
Asian	14.37	11.04	15.2	15.48	13.87
Black	17.37	26.18	15.17	23.42	14.66
Hispanic	21.84	28.78	20.1	26.46	19.76
Other	8.51	7.44	8.78	7.22	9.09
Language					
English	74.54	70.47	75.56	69.37	76.86
Non-English	25.46	29.53	24.44	30.63	23.14
SES Index Quintile					
First	14.91	20.72	13.46	20.45	12.43
Second	16.70	22.21	15.32	19.89	15.27
Third	18.54	18.73	18.49	18.93	18.36
Fourth	19.68	17.74	20.16	18.44	20.23
Fifth	30.17	20.60	32.57	22.29	33.70
Marital Status					
Married	70.62	60.55	73.14	64.55	73.34
Unmarried	29.38	39.45	26.86	35.45	26.66
Maternal Age (Years), mean (SD)	28.53 (6.33)	27.38 (6.1)	28.82 (6.36)	27.93 (6.35)	28.8 (6.31)
Neighborhood Deprivation Index, mean (SD)	−0.12 (1.05)	0.47 (1.08)	−0.27 (0.99)	0.09 (1.09)	−0.22 (1.02)

**Table 2:** Crude and adjusted ^1^ parameter estimates for associations between high isophorone exposure and math scale scores among the subset of the ECLS-B 2001 birth cohort living in urban communities (N = 4050).

Parameter	Crude	Adjusted ^1^
	Estimate [95% CI]	Estimate [95% CI]
Intercept	42.00 [41.30, 42.71]	35.67 [32.81, 38.53]
Isophorone		
≤0.49 ng/m^3^	Reference	Reference
>0.49 ng/m^3^	−3.81 [−5.12, −2.51]	−1.63 [−2.91, −0.34]
Race		
White		Reference
Black		−2.61 [−3.92, −1.31]
Hispanic		−1.90 [−3.72, −0.08]
Asian		3.01 [1.47, 4.55]
Other		−1.53 [−3.88, 0.83]
Maternal Age		0.09 [0.01, 0.18]
Marital Status		
Married		Reference
Unmarried		−0.73 [−1.89, 0.43]
Language		
English		Reference
Non-English		0.05 [−1.51, 1.62]
SES Index Quintile		
First		Reference
Second		2.43 [0.74, 4.12]
Third		4.13 [2.61, 5.65]
Fourth		5.43 [3.63, 7.24]
Fifth		8.34 [6.34, 10.34]
Neighborhood Deprivation Index		−0.35 [−0.87, 0.18]

^1^ Adjusted models include all shown variables.

**Table 3:** Crude and adjusted ^1^ parameter estimates for associations between low HOME Score and math scale scores among the subset of the ECLS-B 2001 birth cohort living in urban communities (N = 4050).

Parameter	Crude	Adjusted ^1^
	Estimate [95% CI]	Estimate [95% CI]
Intercept	42.14 [41.41, 42.87]	35.74 [32.95, 38.54]
HOME Score		
>9 (out of 12)	Reference	Reference
≤9 (out of 12)	−3.06 [−4.24, −1.89]	−1.20 [−2.30, −0.10]
Race		
White		Reference
Black		−2.67 [−4.03, −1.30]
Hispanic		−1.93 [−3.69, −0.17]
Asian		3.14 [1.63, 4.65]
Other		−1.56 [−3.95, 0.83]
Maternal Age		0.10 [0.01, 0.18]
Marital Status		
Married		Reference
Unmarried		−0.67 [−1.84, 0.51]
Language		
English		Reference
Non-English		−0.01 [−1.54, 1.52]
SES Index Quintile		
First		Reference
Second		2.34 [0.67, 4.01]
Third		4.03 [2.53, 5.53]
Fourth		5.25 [3.47, 7.04]
Fifth		8.12 [6.13, 10.11]
Neighborhood Deprivation Index		−0.46 [−0.94, 0.02]

^1^ Adjusted models include all shown variables.

**Table 4:** Crude and adjusted ^1^ parameter estimates for associations between high isophorone exposure, low HOME score, and math scale scores among the subset of the ECLS-B 2001 birth cohort living in urban communities (N = 4050).

Parameter	Without Interaction		With Interaction	
	Crude	Adjusted ^1^	Crude	Adjusted ^1^
	Estimate [95% CI]	Estimate [95% CI]	Estimate [95% CI]	Estimate [95% CI]
Intercept	42.69 [41.94, 43.44]	35.91 [33.09, 38.72]	42.64 [41.86, 43.43]	35.86 [33.05, 38.68]
Isophorone				
≤0.49 ng/m^3^	Reference	Reference	Reference	Reference
>0.49 ng/m^3^	3.33 [−4.65, −2.00]	−1.48 [−2.79, −0.18]	−3.04 [−4.61, −1.46]	−1.10 [−2.48, 0.28]
HOME Score				
>9 (out of 12)	Reference	Reference	Reference	Reference
≤9 (out of 12)	−2.57 [−3.75, −1.40]	−1.05 [−2.15, 0.05]	−2.40 [−3.72, −1.07]	−0.82 [−2.05, 0.41]
Interaction Term for Isophorone and HOME Score			−0.71 [−3.20, 1.78]	−0.92 [−3.14, 1.29]
Race				
White		Reference		Reference
Black		−2.51 [−3.85, −1.17]		−2.53 [−3.87, −1.19]
Hispanic		−1.82 [−3.62, −0.03]		−1.84 [−3.63, −0.04]
Asian		3.14 [1.61, 4.68]		3.15 [1.61, 4.68]
Other		−1.47 [−3.82, 0.88]		−1.52 [−3.89, 0.85]
Maternal Age		0.09 [0.01, 0.18]		0.09 [0.01, 0.18]
Marital Status				
Married		Reference		Reference
Unmarried		−0.70 [−1.86, 0.47]		−0.69 [−1.86, 0.47]
Language				
English		Reference		Reference
Non-English		0.05 [−1.50, 1.61]		0.06 [−1.49, 1.62]
SES Index Quintile				
First		Reference		Reference
Second		2.41 [0.72, 4.09]		2.37 [0.69, 4.05]
Third		4.09 [2.57, 5.62]		4.08 [2.55, 5.61]
Fourth		5.35 [3.55, 7.15]		5.33 [3.54, 7.12]
Fifth		8.19 [6.19, 10.20]		8.17 [6.17, 10.18]
Neighborhood Deprivation Index		−0.33 [−0.85, 0.19]		−0.33 [−0.85, 0.19]

^1^ Adjusted models include all shown variables.

**Table 5:** Adjusted ^1^ parameter estimates for the association between high isophorone exposure, low HOME score, and math scale scores, stratified by child gender among the subset of the ECLS-B 2001 birth cohort living in urban communities (N = 4050).

Parameter	Males	Females
	Estimate [95% CI]	Estimate [95% CI]
Intercept	34.42 [30.85, 37.98]	37.31 [33.85, 40.77]
Isophorone		
≤0.49 ng/m^3^	Reference	Reference
>0.49 ng/m^3^	−0.86 [−3.18, 1.46]	−1.71 [−3.56, 0.15]
HOME Score		
>9 (out of 12)	Reference	Reference
≤9 (out of 12)	−0.62 [−2.34, 1.09]	−1.07 [−2.55, 0.40]
Interaction Term for Isophorone and HOME Score	−2.37 [−5.87, 1.12]	1.07 [−1.64, 3.79]
Race		
White	Reference	Reference
Black	−2.74 [−4.71, −0.76]	−2.33 [−4.18, −0.47]
Hispanic	−2.00 [−4.71, 0.72]	−1.62 [−3.45, 0.22]
Asian	2.78 [0.35, 5.21]	3.35 [1.34, 5.36]
Other	−2.23 [−5.58, 1.13]	−1.01 [−3.86, 1.83]
Maternal Age	0.09 [−0.02, 0.20]	0.10 [−0.01, 0.21]
Marital Status		
Married	Reference	Reference
Unmarried	−0.19 [−1.83, 1.44]	−1.04 [−2.58, 0.50]
Language		
English		
Non-English	0.88 [−1.48, 3.24]	−0.65 [−2.46, 1.17]
SES Index Quintile		
First	Reference	Reference
Second	2.69 [0.57, 4.81]	1.87 [−0.27, 4.01]
Third	4.88 [2.80, 6.97]	3.29 [1.32, 5.26]
Fourth	6.11 [3.94, 8.28]	4.50 [2.26, 6.74]
Fifth	9.72 [7.21, 12.22]	6.58 [3.90, 9.26]
Neighborhood Deprivation Index	−0.19 [−1.01, 0.63]	−0.49 [−1.17, 0.18]

^1^ Models include all shown variables.
